# Fabrication and Underwater Testing of a Vector Hydrophone Comprising a Triaxial Piezoelectric Accelerometer and Spherical Hydrophone

**DOI:** 10.3390/s22249796

**Published:** 2022-12-13

**Authors:** Taehoun Roh, Hong Goo Yeo, Cheeyoung Joh, Yongrae Roh, Kyungseop Kim, Hee-seon Seo, Hongsoo Choi

**Affiliations:** 1 Department of Robotics Engineering, DGIST, Daegu 42988, Republic of Korea; 2 Department of Advanced Materials Engineering, Sun Moon University Asan, Asan 31460, Republic of Korea; 3 School of Mechanical Engineering, Kyungpook National University, Daegu 41566, Republic of Korea; 4 Agency for Defense Development, Changwon 51678, Republic of Korea

**Keywords:** piezoelectric accelerometer, PMN-PT piezoelectric single crystal, vector hydrophone

## Abstract

A vector hydrophone is an underwater acoustic sensor that can detect the direction of a sound source. Wide-band characteristics and high sensitivity enhance the performance of underwater surveillance systems in complex environments. A vector hydrophone comprising a triaxial piezoelectric accelerometer and spherical hydrophone was fabricated and tested in the air and underwater. The vector hydrophone was designed to exceed the quantitative figures of merit (i.e., receiving voltage sensitivity and bandwidth) of commercially available hydrophones. Accelerometer performance was enhanced by placing a pair of piezoelectric single crystals on each axis and modifying the seismic mass material. The receiving voltage sensitivity of the omnidirectional hydrophone was approximately −160 dB relative to 1 V/μPa with the amplifier in water; the sensitivity of the accelerometer exceeded 300 mV/g in air and −215 dB relative to 1 V/μPa underwater over the frequency range of interest. The receiving directivity of the vector hydrophone was validated underwater, which confirmed that it could detect the direction of a sound source.

## 1. Introduction

A vector hydrophone is an underwater acoustic sensor that can detect the azimuth and elevation angle of a sound source in water [1]. The traditional way to detect the direction of a sound source is to use spatially distributed sensors [2,3] such as the towed array sonar system (TASS), which consists of omnidirectional hydrophones. However, due to the symmetric placement of the sensors along the axis, TASS suffers from a left–right ambiguity issue. This is solved by maneuvering the ship or using multiple sonar arrays, which make the system expensive and complex [4,5,6].

Leslie et al. developed another method to implement a vector hydrophone by combining a pressure sensor with a particle velocity sensor [7]. This type of vector hydrophone combines a dipole receiving beam pattern from the vector sensor and an omnidirectional receiving beam pattern from the scalar hydrophone to form a cardioid beam pattern [8]. The vector sensor for this purpose can be implemented in various ways to meet specific requirements [9]. Wilcoxon developed a three-dimensional (3D) vector hydrophone for the US Navy using a lead zirconate titanate (PZT) ring hydrophone and a triaxial accelerometer with a four-channel amplifier. In that research, the accelerometer displayed a dipole pattern up to 10 kHz, but the internal structure of the accelerometer was not disclosed [10,11]. Another type of vector hydrophone is the cilia-type MEMS. The bio-inspired MEMS structure, which resembles the neuromast of a fish, is used to detect the particle velocity of water [12,13,14]. Another vector hydrophone is implemented using a multimode vector hydrophone. Lim et al. developed a multimode ring vector hydrophone by dividing a PZT ring hydrophone and validated its operation underwater [15,16]. In 2021, Hongkun et al. developed an inertial-type vector hydrophone for an airborne sonobuoy. They integrated a biaxial accelerometer with a magnetic compass and signal conditioning circuits to achieve a receiving voltage sensitivity of −157 dB over an operating frequency range from 5 Hz to 2.5 kHz [17]. Cho et al. developed a miniaturized device involving a cylindrical pressure-sensing hydrophone and an orthogonally placed cantilever-type accelerometer that detected 2D sounds. The receiving voltage sensitivities of the accelerometer and hydrophone were −199 and −196 dB, respectively, at 3 kHz [18]. However, as underwater acoustic techniques have developed, the noise emitted from underwater targets such as torpedoes or submarines has diminished, thus deteriorating the performance of traditional sonar systems. Underwater sensor networking is being studied to overcome this issue. However, a recent sensor network has encountered bandwidth limitations and maintenance issues [19]. A simple vector hydrophone with a wide bandwidth is desirable in underwater networks. To utilize the benefits of an underwater sensor network, the sensor nodes should be inexpensive and operate with a wide bandwidth [20,21]. Mudiyala et al. developed a dual-layer structure for a cymbal transducer to achieve wider bandwidth [22]. Yeo et al. developed a compressive-type vector sensor using a piezoelectric single crystal for ease of fabrication. The fabricated vector sensor was able to form a dipole beam pattern, suggesting that it could be used as an underwater vector sensor [23].

**Figure 1:**
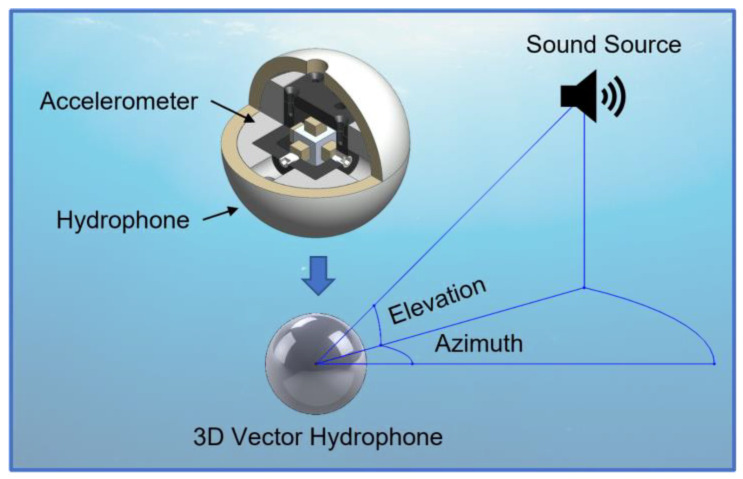
Schematic illustration of a three-dimensional vector hydrophone equipped with a triaxial accelerometer.

We present a 3D vector hydrophone with three accelerometers operating in the x, y, and z directions. We use piezoelectric single-crystal triaxial accelerometers to ensure reliable assembly while maintaining high sensitivity and broadband characteristics (Figure 1). The two sets of lead magnesium niobate-lead titanate (PMN-PT) piezoelectric single crystals in each unidirectional accelerometer increase the hydrophone sensitivity and bandwidth, but they reduce the size and weight. Furthermore, structural symmetry is achieved by distributing the seismic mass along all three axes and placing a pair of PMN-PT crystals on each axis. Thus, reduced off-axis sensitivity and neutralization of the output signal by deformation are obtained. The assembly of the three accelerometers in a metallic cube inside the spherical hydrophone is easier and more reliable than our previous method [23]. The fabricated triaxial accelerometer was characterized in the air using the back-to-back method, and the directivity of the accelerometer was validated using a shaker-mounted goniometer. The vector hydrophone was then assembled and characterized underwater to measure the receiving voltage sensitivity and directivity.

## 2. Design and Fabrication

### 2.1. Spherical Hydrophone

**Figure 2:**
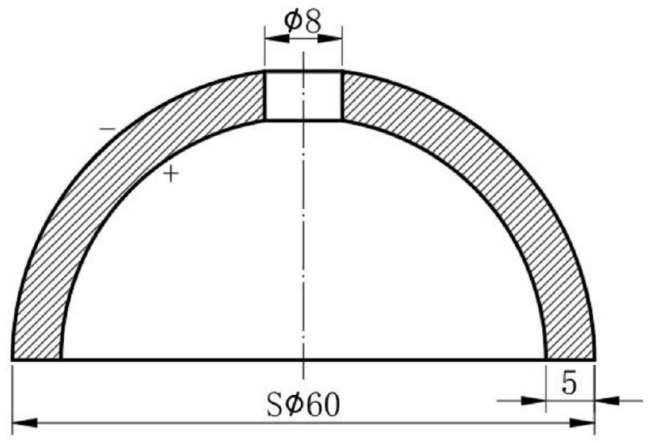
Dimensions of the thin-shelled hemispherical piezoelectric structure.

The simple structure and flat response of two thin-shelled hemispherical piezoelectric ceramics (Shenzhen Hurricane Tech. Co., Ltd., Shenzhen, Guangdong, China) enabled the measurement of the omnidirectional incident sound pressure [24]. A pair of hemispherical hydrophones was designed with a wall thickness of 5 mm, an outer diameter of 60 mm, and an 8 mm diameter hole in the top to meet the specific requirements of receiving voltage sensitivity and to accommodate the 3D accelerometer within the spherical hydrophone (Figure 2). Two silver electrodes were electroplated on the inner and outer surfaces of the hemispherical hydrophones after polishing. The spherical hydrophone was then radially polarized. The electrodes of the hemispherical hydrophones were connected in parallel via direct wire soldering. Holes in both hemispheres were closed with epoxy as the final step of assembly. PZT was used as the material of the spherical hydrophone. The specification of the hemisphere and characteristics of the PZT-43 provided by the manufacturer are shown in Table 1 and Table 2.

### 2.2. Triaxial Piezoelectric Accelerometer

**Figure 3:**
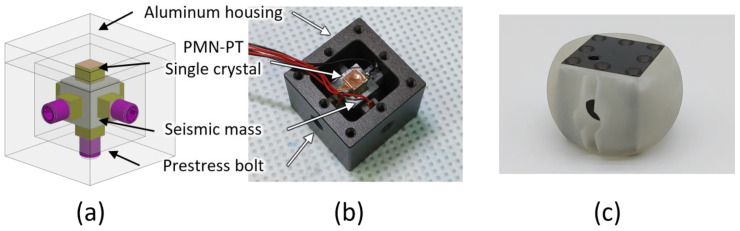
(**a**) Schematic view of the three-dimensional (3D) accelerometer. (**b**) Photograph of the accelerometer without the lid. (**c**) Photograph of the assembled 3D accelerometer in its 3D printed housing.

A compression-type, piezoelectric triaxial accelerometer was used to obtain dipole beam patterns along the three axes (Figure 3). The metallic accelerometer housing was a hollow aluminum cube with a wall thickness of 5 mm and a volume of 28.7 × 28.7 × 28.7 mm^3^. We used three set screws (one per direction) to assemble the seismic mass and the PMN-PT crystals. The cube was hard-anodized; this electrical insulation prevented short circuits. The cube was closed with a lid after the 3D accelerometer had been assembled. A tapped hole was created on three sides of the cube to receive screws that applied preloads during the assembly of PMN-PT crystals. For comparison, cubic seismic masses of tungsten alloy and stainless steel with volumes of 9.5 × 9.5 × 9.5 mm^3^ were also fabricated.

A PMN-PT piezoelectric single crystal (iBULe Photonics Co., Ltd., Incheon, Republic of Korea) was used as the sensing element because of its high piezoelectric coefficient (Table 3). The crystal was fabricated according to the Bridgman method to have a [001] poling direction and was cut into 5 × 5 × 4 mm^3^ cubes. The electrical properties of the crystals provided by the manufacturer are listed in Table 4. Since the crystals had a low phase transition temperature of about 90 °C, direct contact of the soldering iron with the crystal was avoided. Accordingly, the wires were soldered onto copper tape, which was then attached to the crystals for wiring. Polyimide tape was attached to the opposite side of the adhesive side of the copper tape for insulation. The crystals with the copper electrode tapes were attached to the cubic seismic mass using cyanoacrylate glue (Loctite 401; Henkel, Düsseldorf, Germany). The assembled structure was then placed in the cube and clamped in place using the set screws.

**Figure 4:**
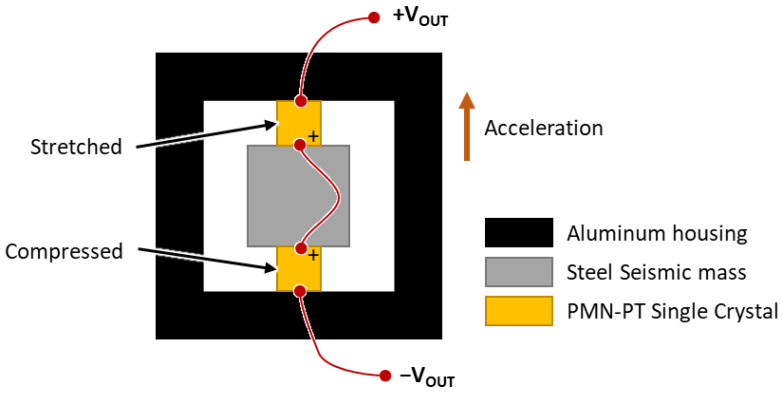
Operating principle of a compressive-type piezoelectric accelerometer with a pair of PMN-PT single crystals (other axes are omitted for clarity). The plus symbol on a single crystal indicates positive voltage output under compression.

In contrast to conventional compression-type piezoelectric accelerometers, we placed two PMN-PT crystals per axis to increase sensitivity and ensure structural symmetry. The poling directions of the crystals were faced, and the electrodes were connected in series to increase the output voltage of the accelerometer. During the assembly of the three accelerometers in the hollow aluminum cube, the set screws were adjusted such that the resonant frequencies of each axis were similar when measured by an impedance analyzer (HP4294A; Agilent, Santa Clara, CA, USA). In the new configuration, stress release increases upon acceleration, and voltage output upon deformation of the aluminum cube is neutralized (Figure 4).

### 2.3. Assembly of the 3D Vector Hydrophone

**Figure 5:**
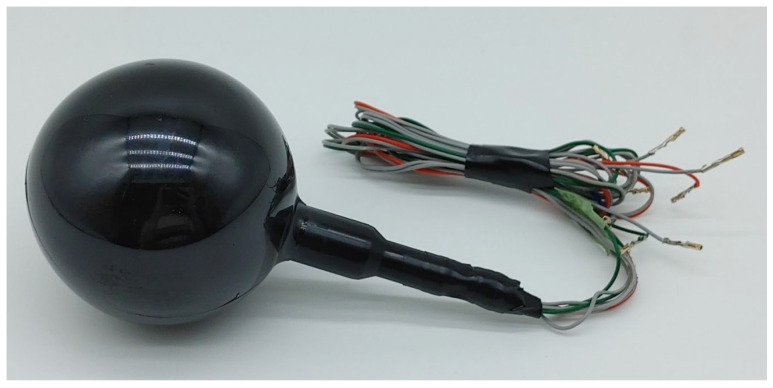
The molded three-dimensional hydrophone.

The triaxial accelerometer was placed in the spherical hydrophone after fabrication and characterization of its sensitivity and directivity in air. The spherical housing was fabricated using a 3D printer (ProJet MJP 5500X; 3D Systems, York County, SC, USA) to fill the gap between the accelerometer and spherical hydrophone (Figure 3c). The volume of the housing was maximized to increase structural integrity and leave minimal space for wiring. Then, the signal wires were attached. The gap between the accelerometer and housing was filled with commercial two-part epoxy (Power Epoxy; USCHEM, Seoul, Republic of Korea). The same epoxy was then used to assemble the housing with a pair of hemispherical PZT hydrophones. After assembly, the vector hydrophone was coated with a 4 mm thick polyurethane layer using a soft mold to ensure the isolation of the outer electrode surface from the water (Young Sang Polytech, Incheon, Republic of Korea) as shown in Figure 5.

## 3. Characterization of the Accelerometer

### 3.1. Sensitivity Measurement in Air

**Figure 6:**
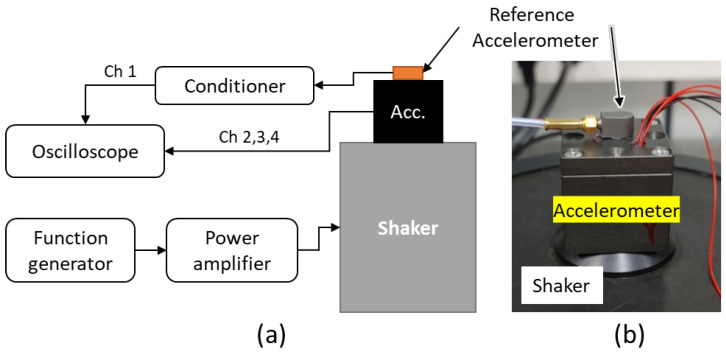
(**a**) Schematic illustration and (**b**) photograph of the sensitivity measurement setup.

The sensitivity response of the accelerometer to frequency was measured prior to its integration with the spherical hydrophone because the assembly was irreversible. Measurements were performed using a back-to-back method that involved a reference accelerometer (352A59; PCB Piezotronics, Depew, NY, USA) with a known sensitivity of 10 mV/g (Figure 6). Due to the limited surface area of the reference accelerometer, it was placed on top of the proposed triaxial accelerometer. Both sensors were glued to the shaker using cyanoacrylate adhesive and excited together. The measurement was performed three times in each direction; the triaxial accelerometer and reference accelerometer were then detached from the exciter and reglued to measure the other axes. The adhesive residues were removed before gluing so that the thickness of the contact surface remained constant.

**Figure 7:**
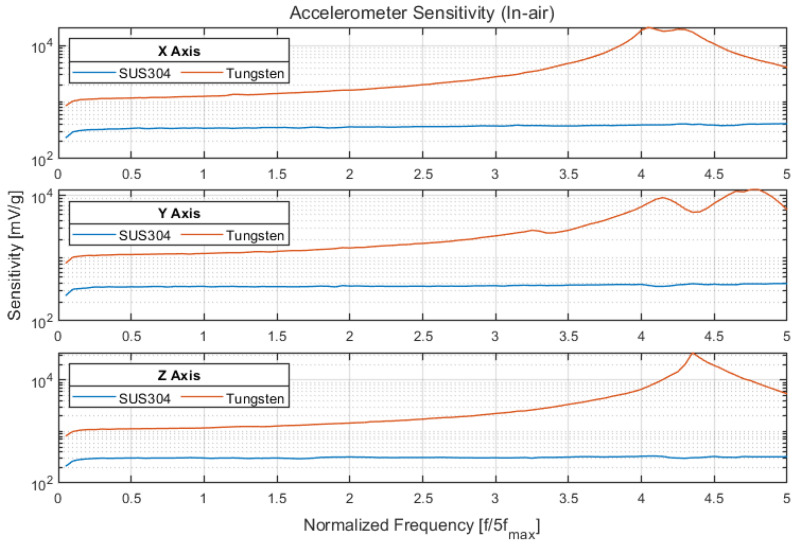
Sensitivity of accelerometer with a stainless-steel or tungsten alloy seismic mass.

The sensitivity of the triaxial accelerometer was measured in conjunction with a tungsten alloy or stainless-steel (SUS403) seismic mass for comparison. The dimension, density, and measured mass of the seismic mass are provided in Table 5, and the sensitivity measurement results are presented in Figure 7. The frequency values (*x* axis) were normalized by 1/5 of the maximum frequency of interest (f_max_) according to the funding agency’s requirement. The acceleration sensitivity ($M_{a}$) is given by Equation (1), where $V_{out}$ is the amplitude of the signal from the accelerometer, and a is the acceleration imparted to the accelerometer (as measured by the reference accelerometer) [23].

(1) $$M_{a}=V_{out}/a$$

The triaxial accelerometer with the tungsten alloy seismic mass exhibited a sensitivity of 1100–1300 mV/g at the normalized frequency, along with a peak within the frequency range of interest; thus, it was inappropriate for use over the entire frequency range. However, the triaxial accelerometer with the stainless-steel seismic mass exhibited a flat response of 300–350 mV/g over the entire frequency range of interest; it lacked a specific peak.

### 3.2. Directivity Measurement in Air

**Figure 8:**
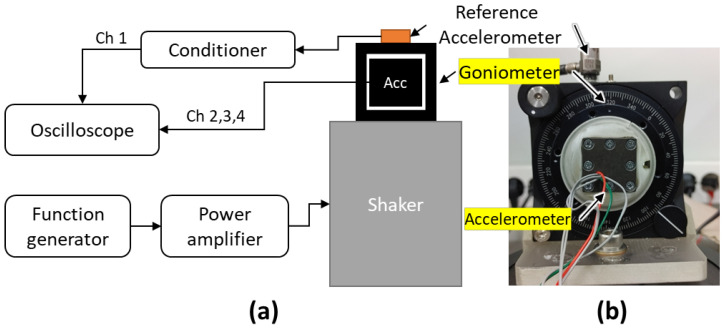
(**a**) Schematic illustration and (**b**) photograph of the directivity measurement setup.

The directivity of the triaxial accelerometer was also measured in the air prior to assembly. For this measurement, a goniometer was mounted on the shaker, and the triaxial accelerometer was clamped in the goniometer using a 3D-printed jig (Figure 8). The measurement was performed at a specific frequency where the acceleration of the goniometer monitored by the reference accelerometer was not distorted. The reference accelerometer was used primarily to monitor the acceleration of the goniometer and also to mimic the spherical hydrophone’s omnidirectional signal so that it could be used to form a cardioid pattern in further research.

**Figure 9:**
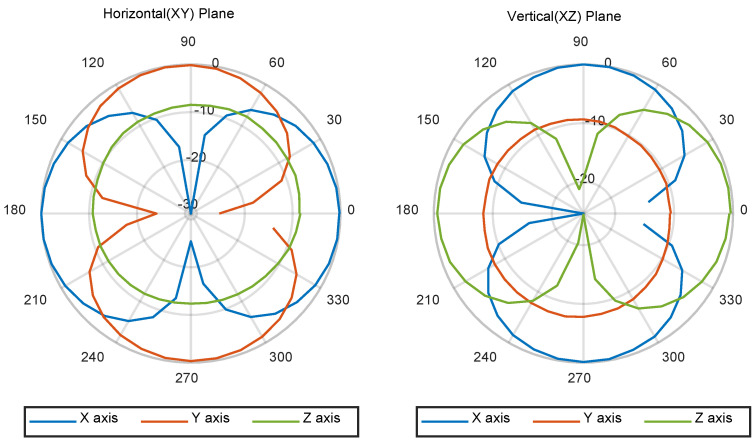
Measured directivities obtained from the goniometer experiment in air.

The voltage from the accelerometer was measured; the directivity was then calculated using Equation (2), as shown in Figure 9. Since the accelerometer could only be rotated about one axis, the directivity measurement was performed in both the horizontal and vertical planes. The measurement was performed over 360° with a step angle of 10°. Then, the amplitude of the voltage output at each angle was measured and normalized by the maximum amplitude of output voltages measured in each setup, i.e., in the horizontal and vertical configurations, and then plotted. The test frequency was 0.6-fold of the normalized frequency. Under this condition, the acceleration of the goniometer, as monitored by the reference accelerometer, was not distorted. At a test frequency in excess of 0.8-fold of the normalized frequency, the experimental setup became unstable in that the signal amplitude changed each time the angle of the goniometer was adjusted. The *x* and *y* axes both displayed a dipole pattern, while the *z* axis showed omnidirectional sensitivity because the *z* axis was orthogonal to the acceleration axis at any angle. For the vertical (XZ) direction, the *x* and *z* axes displayed a dipole pattern, while the *y* axis showed an omnidirectional response, as expected. It was also confirmed that the pair of dipole patterns was orthogonal in the horizontal and vertical directivity measurements.

(2) $$Directiviy=20\log_{10}\left(V_{out}/V_{max}\right)\;\left[dB\right]$$

## 4. Underwater Characterization of the 3D Vector Hydrophone

**Figure 10:**
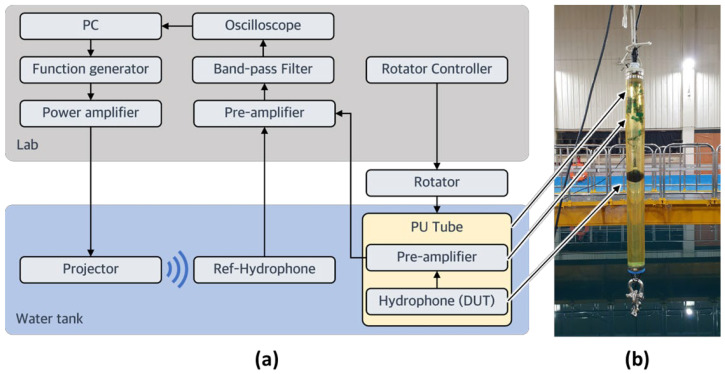
(**a**) Schematic flow diagram of the underwater experiment. (**b**) Photograph of the prototype three-dimensional vector hydrophone in the polyurethane tube.

After the triaxial accelerometer was characterized in air, it was assembled with a spherical hydrophone to fabricate the 3D vector hydrophone, which was characterized in a water tank. The device was submerged with in-house-built preamplifiers, which provided amplification of 21.6 dB. A polyurethane tube filled with oil was used to insulate the connection between the 3D vector hydrophone and preamplifiers. Then, the signals were further amplified (10 dB of amplification) using a signal conditioner (VP2000; Teledyne, Thousand Oaks, CA, USA). Finally, a bandpass filter (model 3944; Krohn-Hite, Brockton, MA, USA) located at the end of the wires was used to reduce the noise. The signals were then measured using an oscilloscope connected to a computer equipped with analysis software. In the underwater test, the receiving voltage sensitivity was measured, and the ability to form a cardioid beam pattern was validated. The measurement setup is illustrated in Figure 10. The projector and the reference and vector hydrophones were submerged to a depth of 4.96 m. The distances from the projector to the reference and vector hydrophones were 4.34 and 4.1 m, respectively. For directivity measurements, a rotator was used to rotate the polyurethane tube containing the vector hydrophone. For the acoustic wave transmission measurement, a software-controlled function generator was used to generate a tone burst signal with a specific frequency and specific cycles. The generated signals were amplified by a power amplifier and then radiated from an underwater projector (D/11; Neptune Sonar, Kelk, UK). A reference hydrophone (TC4032; Teledyne) with a receiving voltage sensitivity of −170 dB was used to monitor the acoustic wave distortion and reflection; it was also used to calibrate the receiving voltage sensitivity of our vector hydrophone.

**Figure 11:**
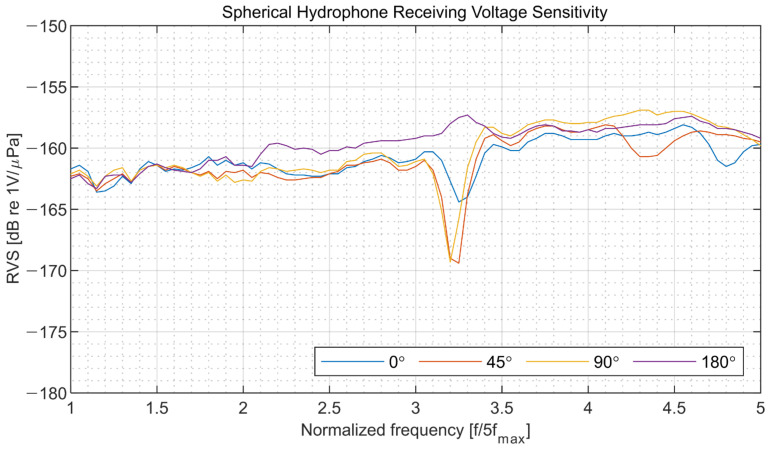
Measured receiving voltage sensitivity (RVS) of the spherical hydrophone with 31.6 dB amplification.

**Figure 12:**
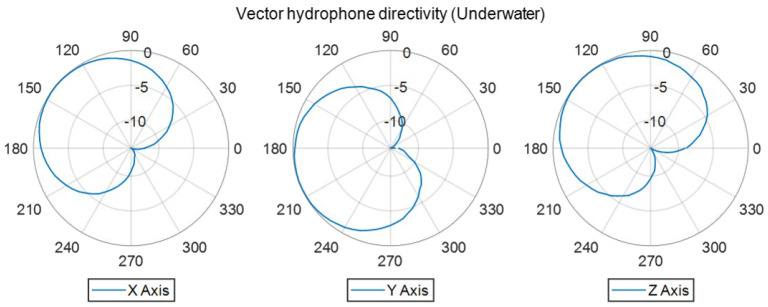
Cardioid patterns were measured underwater at a normalized frequency of 2.5.

**Figure 13:**
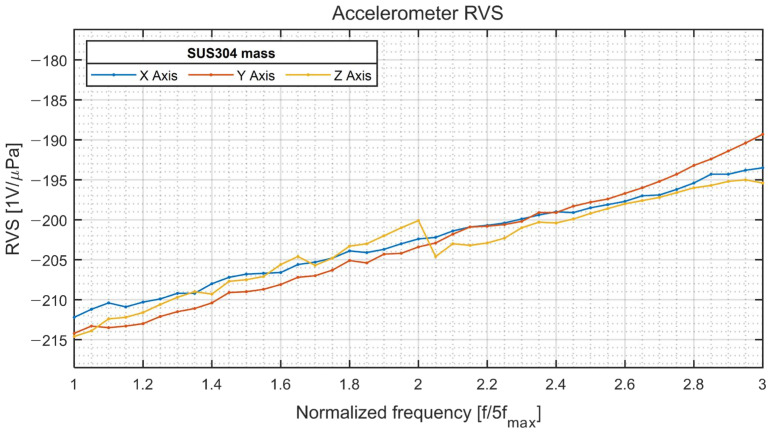
Underwater receptor voltage sensitivity of the accelerometer.

The results of the underwater measurements are presented in Figure 11, Figure 12 and Figure 13. Spherical hydrophone receiving voltage sensitivity measurements were performed four times during each revolution at a step angle of 90° to validate omnidirectionality. The pressure sensitivity ($M_{p}$) (in dB) was calculated using Equations (3) and (4), where $V\;\mathrm{and}\;P_{i}$ denote the output voltage and incident acoustic pressure, respectively.

(3) $$M_{p}=V_{out}/P_{i}\left[V/\mathrm{Pa}\right]$$

(4) $$RVS=20\log_{10}\left(M_{p}/10^{-6}\right)\left[\mathrm{dB}\;re\;1V/\mu \mathrm{Pa}\right]$$

At a normalized frequency of approximately 3.25 (Figure 11), a sharp drop of −10 dB in sensitivity was observed, possibly caused by a feature of the contact boundary between the two hemispherical hydrophones used to make the spherical hydrophone or by an interaction between the 3D accelerometer and the hydrophone. For the 3D vector hydrophone directivity measurement, the hydrophone was rotated using the rotator along a horizontal plane and a vertical plane. The signals were acquired during one revolution at a step angle of 5°. The signals captured from the spherical hydrophone and triaxial accelerometer in each direction were normalized by their maximum output amplitude and delay so that they could be combined to build a cardioid receiving pattern (Figure 12). The directivities along the *x* and *y* axes were orthogonal since both signals could be obtained with the same horizontal measurement setup. The *z* axis displayed a cardioid-shaped receiving pattern as well. Figure 13 shows that the receiving voltage sensitivity of the accelerometer increased linearly; the sensitivities along all axes were similar.

## 5. Conclusions

A 3D vector hydrophone consisting of a spherical hydrophone and piezoelectric triaxial accelerometer was designed and fabricated. A pair of thin-shelled hemispherical piezoelectric structures were used to fabricate a spherical omnidirectional hydrophone with a wall thickness of 5 mm, an outer diameter of 60 mm, and an 8 mm diameter hole in the top. The spherical PZT hydrophone was radially polarized. A compression-type piezoelectric triaxial accelerometer was designed and fabricated with PMN-PT single crystals as the sensing element due to their high piezoelectric coefficient. Each PMN-PT crystal had a [001] poling direction and was cut into 5 × 5 × 4 mm^3^ cubes. The fabricated accelerometer showed a flat response over a wide frequency range and clear directivity when tested with a stainless-steel seismic mass in the air. The accelerometer was placed in the spherical hydrophone to realize a 3D vector hydrophone, which was then coated with a 4 mm thick polyurethane layer for waterproofing. The assembled spherical hydrophone was calibrated underwater and showed a flat response across the frequency range of interest. The vector hydrophone also showed cardioid patterns along three axes, demonstrating that it could be used to detect the direction of the sound source.

## Figures and Tables

**Table 1:** Electromechanical properties of the piezoceramic hemisphere.

Parameter	Unit	Value
Resonant frequency (Fr)	kHz	36.5 ± 2.5
Bandwidth (Δf)	kHz	3.8
Capacitance (C)	pf	11,700 ± 15%

**Table 2:** Material properties of PZT-43 used for the PZT hemispherical hydrophone.

Electromechanical coupling coefficient	k_p_	0.58	Piezoelectric charge constant (10^−12^ m/V)	d_31_	−138
	k_31_	0.34		d_33_	300
	k_33_	0.68	Piezoelectric voltage constant (10^−3^ Vm/N)	g_31_	11
	k_t_	0.48		g_33_	24
Free dielectric constant	$\epsilon_{r3}^{T}$	1420	Sound wave velocity (m/s)	$V_{d}$	3360
Dielectric dissipation factor	tan*δ*	0.5		$V_{1}$	3200
Elastic compliance constant	$S_{11}^{e}$	13.2		$V_{3}$	3750
Mechanical quality factor	$Q_{m}$	600		Vt	4150
Young’s modulus (10^9^ N/m^2^)	$Y_{11}^{E}$	76	Curie temperature (°C)	$T_{c}$	320
Density (g/m^3^)	ρ	7500	Poisson’s ratio	σE	0.30

**Table 3:** Material properties of PMN-PT piezoelectric single crystals [25]. (Copyright granted by iBULe Photonics Inc., Incheon, Republic of Korea).

Parameter	Symbol	Units	[001] Poled	
			Low PT	High PT
Relative dielectric constant	$\epsilon_{33}^{T}/\epsilon_{0}$	-	4842	7000
Piezoelectric constant	$d_{\mathrm{ij}}$	×10^−12^ C/N	$d_{33}\;$= 1282	$d_{33}$ = 1620
Elastic compliance	$S_{ij}^{E}$	×10^−12^ m^2^/N	$S_{33}^{E}$ = 47	$S_{33}^{E}$ = 56
Curie temperature	Trt	°C	95	85
Coercive field	$E_{C}$	kV/cm	2	2.5
Density	ρ	kg/m^3^	8080	

**Table 4:** Characteristics of fabricated PMN-PT piezoelectric single crystals.

Parameter	Symbol	Units	
Dimensions	–	mm	5 × 5 × 4
Capacitance	$C_{3}^{T}$	nF	0.249–0.278
Dielectric dissipation factor	tanδ	%	0.11–0.31
Resonance frequency	$f_{r}$	kHz	155.50–163.00
Antiresonance frequency	$f_{a}$	kHz	237.25–243.00
Free dielectric constant	$\epsilon_{r}^{T}$	–	4506–5030

**Table 5:** Properties of tungsten alloy and stainless-steel seismic masses.

Parameter	Units	Material	
		Tungsten Alloy	Stainless Steel
Dimensions	mm	9.5 × 9.5 × 9.5	
Density	g/cm^3^	19.25	7.93
Mass (measured)	g	19.68	6.46

## Data Availability

Not applicable.
